# The Ca^2+^/Calcineurin-Dependent Signaling Pathway in the Gray Mold *Botrytis cinerea*: The Role of Calcipressin in Modulating Calcineurin Activity

**DOI:** 10.1371/journal.pone.0041761

**Published:** 2012-07-23

**Authors:** Karin Harren, Julia Schumacher, Bettina Tudzynski

**Affiliations:** Westfälische Wilhelms-Universität Münster, Institute of Biology and Biotechnology of Plants, Münster, Germany; University of Wisconsin - Madison, United States of America

## Abstract

In the gray mold fungus *Botrytis cinerea* the Gα subunit Bcg1 of a heterotrimeric G protein is an upstream activator of the Ca^2+^/calmodulin-dependent phosphatase calcineurin. In this study we focused on the functional characterization of the catalytic subunit of calcineurin (BcCnA) and its putative regulator calcipressin (BcRcn1). We deleted the genes encoding both proteins to examine their role concerning growth, differentiation and virulence. The Δ*bccnA* mutant shows a severe growth defect, does not produce conidia and is avirulent, while the loss of BcRcn1 caused retardation of hyphal growth and delayed infection of host plants, but had no impact on conidiation and sclerotia formation. Expression of several calcineurin-dependent genes and *bccnA* itself is positively affected by BcRcn1. Complementation of the Δ*bcrcn1* mutant with a GFP-BcRcn1 fusion construct revealed that BcRcn1 is localized in the cytoplasm and accumulates around the nuclei. Furthermore, we showed that BcCnA physically interacts with BcRcn1 and the regulatory subunit of calcineurin, BcCnB. We investigated the impact of several protein domains characteristic for modulation and activation of BcCnA via BcRcn1, such as the phosphorylation sites and the calcineurin-docking site, by physical interaction studies between BcCnA and wild-type and mutated copies of BcRcn1. Based on the observed phenotypes we conclude that BcRcn1 acts as a positive modulator of BcCnA and the Ca^2+^/calcineurin-mediated signal transduction in *B. cinerea*, and that both proteins regulate fungal development and virulence.

## Introduction

The necrotrophic ascomycete *Botrytis cinerea* is able to infect a broad range of dicotyledonous host plants including economically important vegetable and fruit crops such as tomatoes, beans, grape vine or strawberries [1], [2]. The filamentous fungus is both a serious pathogen causing economically significant losses in agriculture, and an ideal model organism to study pathogen-host interactions. The recently published genome sequences of the two different *B. cinerea* strains B05.10 and T4 [3] makes application of molecular techniques like knock-out approaches and high through-put genomic tools much easier and stimulates research on this plant pathogen.

The fine-tuned regulation of all processes of life in fungal plant and human pathogens, such as growth, development, morphological differentiation and host infection, is essential for survival [4]–[8]. Fungi are able to sense changes in the environment, and to respond appropriately by cellular changes on transcript and protein levels. Components of signaling pathways, such as mitogen-activated protein kinase (MAPK) cascades, the adenylate cyclase/cyclic AMP (cAMP)/protein kinase A cascade and the calcium/calcineurin pathway, which are highly conserved in yeast, filamentous fungi and even higher eukaryotes, control fundamental aspects of fungal growth, development and reproduction [9]. However, their targets and biological functions may differ and require study in each organism.

Calcineurin (CN) is a highly conserved Ca^2+^/calmodulin-regulated type 2B protein phosphatase that is crucial for mediating cellular stress responses. Stress-induced transient increases of intracellular Ca^2+^-concentrations from either intracellular stores or extracellular sources are sensed by calmodulin (CaM), a small protein containing four EF-hand motifs for binding Ca^2+^ ions. The Ca^2+^-CaM complex then activates various target proteins including protein kinases and the phosphatase CN. Functional CN consists in its inactivated form of two subunits, a catalytic (CNA) and a regulatory subunit (CNB) [10]–[14]. CN differs from the so-called CN-like phosphatases in its C-terminal extension which contains domains important for regulation of enzymatic activity (autoinhibitory domain, AID) and for the interaction with the two regulatory proteins CNB and CaM. Activation occurs when the cytosolic Ca^2+^ level is increased and free Ca^2+^ is bound by CaM and CNB [15]. Ca^2+^-bound CaM interacts with the CaM-binding domain of CNA, releasing the autoinhibitory domain (AID) from the substrate-binding pocket of the catalytic domain by a conformational change that relieves autoinhibition and leads to activation of the phosphatase and dephosphorylation of CN substrates [13].

One of the well known CN substrates is the conserved zinc-finger transcription factor Crz1 which is translocated to the nucleus to regulate expression of target genes, e.g. those involved in cell wall integrity and ion homeostasis [16]. In *Saccharomyces cerevisiae* [10], [17]–[19] and *Schizosaccharomyces pombe* [20], the importance of CN has been established in various stress responses including high temperature, high concentrations of ions, cell wall stress and exposure to mating pheromone. In the human pathogens *Candida albicans*, *Candida dubliniensis* and *Aspergillus fumigatus*, the CN-dependent cascade controls cell wall integrity, stress resistance and response, morphogenesis, serum survival and virulence [4], [21]–[26]. For *C. albicans* it has also been shown that CN is a key mediator of Hsp90-dependent azole resistance [6]. In plant pathogenic fungi such as *Aspergillus oryzae, Magnaporthe oryzae, Ustilago maydis* and *Ustilago hordei,* a role for CN was not only suggested in hyphal growth, but also in environmental stress adaptation, e.g. at alkaline pH or high NaCl concentrations, during formation of conidia and appressoria, for sexual development, cell wall integrity, and virulence [27]. Furthermore, in *S. cerevisiae* and *U. maydis* CN was shown to be a putative antagonist of the protein kinase A (PKA) demonstrating a connection between the CN- and the cAMP-dependent cascades [28], [29].

In *B. cinerea*, the impact of CN on cellular processes was first studied by Viaud et al. [30] using an inhibitor of calcineurin, cyclosporine A (CsA). These studies indicated that CN is probably involved in regulation of hyphal morphology, formation of infection structures and secondary metabolism and led to the identification of 18 CN-dependent (CND) genes. Among the CND genes are the botrydial biosynthesis genes which were previously shown to be controlled by the Gα subunit Bcg1 [31], [32] suggesting a connection between both signaling pathways. Later we could confirm this functional link by demonstrating that Bcg1 does not only control the adenylate cyclase/cAMP/PKA cascade [33], but also the Ca^2+^/CN-signaling cascade [34], [35]. Further research demonstrated for the first time in a phytopathogenic fungus that the Gα subunit Bcg1 acts as an upstream-activator of the *B. cinerea* Ca^2+^-signaling cascade via phospholipase C (BcPlc1) [35]. Detailed studies on the *B. cinerea* Ca^2+^-signaling cascade resulted in the identification of the first homolog of the yeast transcription factor Crz1 (CRaZy, calcineurin-responsive zinc finger transcription factor) in a filamentous fungus [34]. BcCrz1 is involved in hyphal morphology, conidiation, sclerotia formation, and stress response. Deletion mutants are hypersensitive to external Ca^2+^ and react when subjected to hypo-osmotic stress. Infection of host plants is significantly retarded in these deletion mutants compared to the wild-type. Interestingly, most of the described phenotypic characteristics could be restored by the addition of Mg^2+^. The subcellular localization of a GFP-BcCrz1 fusion product in yeast cells depends on the Ca^2+^ level and CN activity indicating that CN mediates nuclear translocation of BcCrz1 by its dephosphorylation. The expression of a set of Bcg1- and CN-dependent genes is also affected in Δ*bccrz1* mutants, confirming that this transcription factor acts downstream of CN in *B. cinerea*, as shown in *S. cerevisiae* [34].

Very little is known about regulation of CN activity in *B. cinerea* and other filamentous fungi. In yeast and mammals CN activity is regulated by a family of conserved proteins named “regulators of calcineurin” (RCAN in mammals, RCN in yeast and filamentous fungi) [36]. These proteins, also called calcipressins, have been reported to have both positive and negative effects on CN activity indicating a negative feedback regulation [12], [37]. The Rcn1 homologs Cbp1 (calcineurin-binding protein) and RcnA of the basidiomycete *Cryptococcus neoformans* and the ascomycete *Aspergillus nidulans*, respectively, were shown to bind to CN and modulate CN activity [38], [39]. In *A. fumigatus cbpA* deletion mutants displayed improved Ca^2+^-tolerance, decreased virulence and increased expression of the genes encoding the Ca^2+^/H^+^-exchanger VcxA, the chitin synthase A and CnaA [40].

The function of Rcn1-family proteins is determined by several highly conserved sequence motifs. The stimulatory effect of Rcn1 on CN activity requires phosphorylation of Rcn1 at the conserved FxISPPxSPP motif. In yeast and mammals, Mck1, a Gsk3 protein kinase, phosphorylates Rcn1 at the first serine residue after priming phosphorylation at the second serine residue by a mitogen-activated kinase (MAPK) [37], [41]. The second conserved motif in Rcn1 is a key feature of CN regulation, the CN-specific PxIxIT docking site [42]–[45]. By screening peptide libraries comprising different PxIxIT motif variations of mammalian CN targets and artificial sequences, a high-affinity CN-binding peptide, PVIVIT, was found [46]. Interestingly, a PxIxIT docking site is also present in most studied Crz1 proteins. In yeast, substitution of the native Crz1 sequence with an allele containing the high-affinity PVIVIT sequence resulted in increased survival rates under high salt conditions, but higher sensitivity to alkaline stress. The increased affinity of Crz1 for CN by modification of the PxIxIT motif resulted in a constitutively dephosphorylated and hyperactive transcription factor [47].

In this study we focused on the further characterization of the Ca^2+^/CN-signaling pathway in *B. cinerea* by studying the impact of CN and the calcipressin homolog, BcRcn1, on growth, development and pathogenicity. We identified and manipulated the highly conserved motifs in BcRcn1 and performed interaction studies between BcCnA and BcCnB as well as wild-type and mutated forms of BcRcn1 to elucidate the mechanism of CN regulation in *B. cinerea*.

## Results

### Identification of the *B. cinerea* Calcineurin A Ortholog

A BlastP similarity search in the protein database of *B. cinerea* B05.10 (Broad Institute) using the calcineurin A (CNA1) protein sequence of *S. cerevisiae* as query revealed *bc1g_02606.1* (ABN58724.1) as a potential ortholog (Fig. S1). The predicted open reading frame of *bccnA* contains 1,805 bp including two introns of 60 and 50 bp. The deduced protein has a size of 564 aa and shows a high level of overall amino acid identity to catalytic CN subunits of different organisms, e.g. 98% (E value 0.0) to CNA of *Sclerotinia sclerotiorum*, 85% (0.0) to CNA of *A. fumigatus*, and 60% (0.0) to CNA proteins of mammals.

CNA consists of a catalytic domain homologous to other serine/threonine protein phosphatases (PP2B family) and three regulatory domains at the C-terminus that distinguish CN from other phosphatase family members: the CNB-binding domain, the CaM-binding domain, and the autoinhibitory domain AID. The latter domain binds in the active site cleft in the absence of Ca^2+^/CaM and acts in concert with the CaM-binding domain to confer CaM regulation [13]. All domains were present in BcCnA (Fig. S1). Notably, CNA proteins of filamentous fungi share higher similarities with mammalian proteins than with those of *S. cerevisiae*. Furthermore, we were able to identify the most conserved residues probably responsible for interaction of the immunophilin/immunosuppressant complexes cyclophilin A/CsA and FKBP12/FK506 with the catalytic CN subunit via hydrogen bonds or van der Waals’ interactions.

### Deletion of *bccnA* Results in Severe Growth Defects and Loss of Sporulation and Virulence

It was reported by Viaud et al. [30] that deletion of the catalytic subunit of calcineurin is lethal in *B. cinerea*. However, recently we were able to replace *bccnA* by a resistance cassette (Fig. S2A) using osmotically stabilized selection media for protoplast regeneration. Two independent transformations revealed two mutants with the same drastic phenotype. Homokaryotic Δ*bccnA* mutants were obtained by single spore isolation, and the absence of the *bccnA* wild-type allele was confirmed by PCR (Fig. S2A).

**Figure 1:**
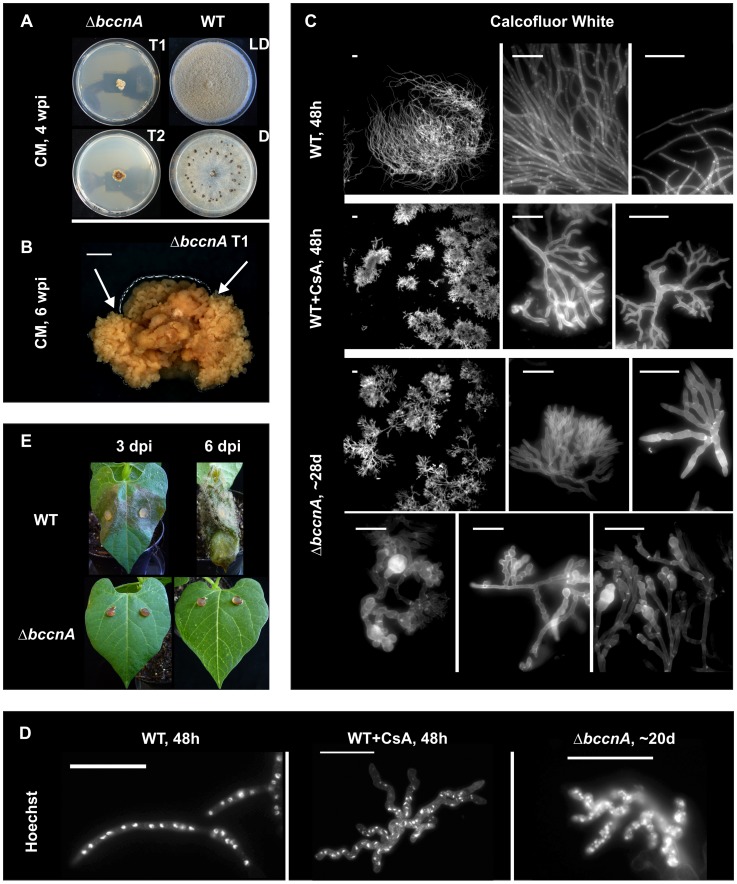
Phenotype of Δ*bccnA* mutants. **A:** Colony growth on CM medium after 4 weeks post inoculation (LD: 12 h rhythm light/dark, D: constant darkness), respectively. Two different primary Δ*bccnA* mutants T1 and T2 were tested in comparison to the *B. cinerea* wild-type B05.10. **B:** Growth of Δ*bccnA* mutant after 6 weeks on CM medium. Arrows indicate sector formation and different morphologies within one colony (scale bar 2 mm). **C–D:** Hyphal morphology and nuclei staining of *B. cinerea* strains with reduced calcineurin activities. Wild-type conidia (WT) were incubated for 48 h in liquid CM medium with and without 10 µg/ml of the calcineurin inhibitor cyclosporine A (CsA). Δ*bccnA* mycelia were incubated for 3 to 4 weeks in liquid CM medium. Cell walls and septa were stained with the fluorescent dye Calcofluor White. Scale bar: 20 µm (C). Nuclei were visualized using the fluorescent dye Hoechst 33342 (for details see Materials and Methods). Scale bar: 50 µm (D). Wild-type conidia (WT) were incubated for 48 h in liquid CM medium with and without 10 µg/ml of the calcineurin inhibitor cyclosporine A (CsA). Δ*bccnA* mycelia were incubated for 4 weeks in liquid CM medium. E: Pathogenicity assay: living bean leaves were inoculated with agar plugs with non-sporulating mycelia of the wild-type (WT) and the Δ*bccnA* mutant. Images were taken 3 and 6 days post infection (dpi).

Both Δ*bccnA* mutants showed a severe growth defect. After several weeks colonies reached diameters of about one to two cm, while the wild-type has a growth rate of one cm per day (Fig. 1A). In addition, a characteristic feature of the mutants is the genetic instability: their appearance changed within weeks from dark-colored to bright-colored colonies. Accordingly, several single spore isolates of the same (heterokaryotic) primary transformant led to colonies exhibiting different phenotypes concerning colony color, growth rate, configuration, and sector formation (Fig. 1B). However, finally the differences between isolates adjusted to one stable phenotype, and after several months all mutants formed small compact bright-colored colonies. In contrast to the wild-type that forms conidia in the light and sclerotia in constant darkness, the mutants formed sclerotia only very rarely after incubation for long periods in constant darkness, whereas conidia were never produced, neither in light nor in the dark.

Microscopic studies showed that Δ*bccnA* mutants exhibit a similar hyphal morphology as the *B. cinerea* wild-type strain B05.10 treated with the CN-inhibitor CsA (Fig. 1C). Addition of CsA to solidified standard medium or spore suspensions results in inhibition of vegetative growth and retardation of conidia germination, respectively. The hyphal morphology was significantly altered: instead of long, steady-growing hyphae in the untreated wild-type control, highly branching mycelia were obtained in the presence of CsA. This increase in hyphal branching was also observed for Δ*bccnA* mutants. The hyphae of Δ*bccnA* mutants were misshaped due to abnormal septation and more frequent branching in addition to the already described slow growth rate. Septation occurred more often in small, irregular intervals, and branches emerge through dichotomous splitting at the hyphal tips. The untreated wild-type nuclei are aligned like pearls in a chain, normally about three to six nuclei per septum section (Fig. 1D). Hyphae of the CsA-treated wild-type strain or the Δ*bccnA* mutant showed similar numbers of nuclei which were arranged almost equispaced. However, as hyphae of these samples are inherently more thickish, not all nuclei are located in a long line, but slightly staggered from each other (Fig. 1D).

To show whether the deletion of *bccnA* has an impact on virulence, wounded and unwounded primary leaves of bean plants were inoculated with agar plugs of the non-sporulating Δ*bccnA* mutant and the wild-type. In contrast to the wild-type, the Δ*bccnA* mutants did not penetrate the bean leaves, neither wounded nor unwounded, and were completely non-virulent (Fig. 1E).

### Expression of a Truncated *bccnA* Copy Lacking the AID Domain

As the deletion of the whole *bccnA* gene resulted in severe defects in fungal growth, development and virulence that make further studies with these mutants extremely difficult, we generated mutants with a truncated *bccnA* gene, *bccnA* ^ΔAID^, controlled by its native promoter but lacking the last 250 bp encoding the AID (Fig. S2A). The *bccnA* wild-type allele was replaced by the truncated *bccnA* ^ΔAID^ allele expecting a mutant with a dominantly active calcineurin A subunit. The transformation resulted in several independent hygromycin-resistant transformants, all showing the homologous integration at the *bccnA* locus (Fig. S2A). Mutants were purified by single spore isolation and screened for absence of the AID domain.

**Figure 2:**
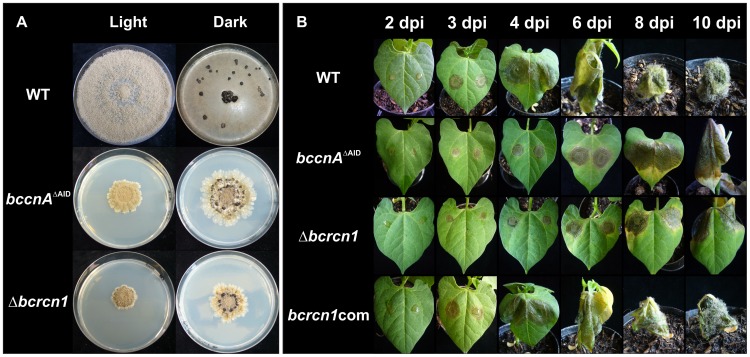
Phenotypical characterization of *bccnA* ^ΔAID^ and *bcrcn1* mutants. Analyzed strains: *B. cinerea* wild-type B05.10 (WT), *bccnA* ^ΔAID^ mutant (expressing truncated version of *bccnA*), Δ*bcrcn1* mutant, Δ*bcrcn1* complementation strain (*bcrcn1*com). A: Growth on complete medium (CM) after 2 weeks in light/dark (12 h/12 h) regime for conidiogenesis and darkness (sclerotia formation). B: Pathogenicity assay: living bean leaves were inoculated with spore suspensions (2*10^5^ spores/ml). Pictures were taken 2–10 days post infection (dpi).

Three homokaryotic *bccnA* ^ΔAID^ mutants displayed severe growth defects on all tested media. On CM medium, they grew as small compact colonies with a significantly reduced growth rate of about 1.5 to 2.5 cm per week. However, in contrast to the Δ*bccnA* mutants, sclerotia formation (constant darkness) and conidiogenesis (light-dark conditions) were unaffected (Fig. 2A). Expression of the truncated bccnA^ΔAID^ gene resulted in reduced virulence: lesion formation on living bean plants was retarded, and in planta conidiation was only rarely observed (Fig. 2B). The retarded infection process was in correlation with the reduced growth rate on solid media, compared to the wild-type.

**Figure 3:**
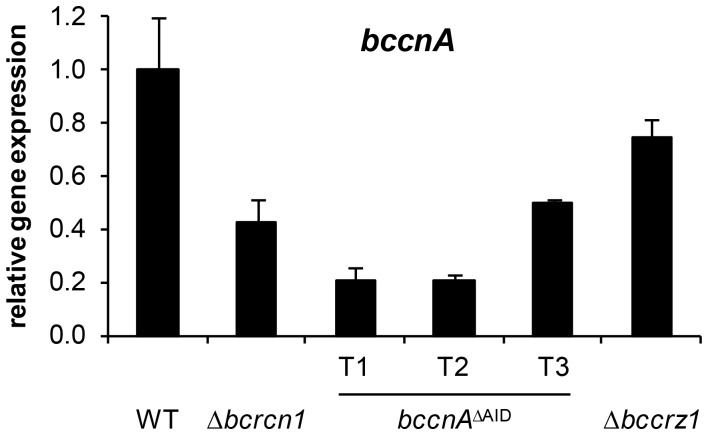
Expression levels of *bccnA* in *B. cinerea* WT, Δ*bcrcn1, bccnA* ^ΔAID^ and Δ*bccrz1* strains detected by quantitative RT-PCR. Expression levels were analyzed in mycelia from liquid cultures (SISLER medium). The quantity of *bccnA* cDNA measured by quantitative RT-PCR was normalized to that of *actA*, *gpd* and *tub* within each strain. The relative abundance of *bccnA* transcript in the wild-type strain B05.10 was assigned to a value of 1 followed by normalization of the other values to this setting. Standard deviations are indicated by error bars.

Surprisingly, the obtained phenotype of the *bccnA* ^ΔAID^ mutants resembled that of the wild-type after inhibition with CsA or FK506 [34]. To explain this unexpected phenotype, expression of the *bccnA* ^ΔAID^ copy in the mutant background was compared with that of the wild-type *bccnA* gene, both controlled by the same native promoter. Interestingly, the transcript level of the truncated gene was undetectable by northern blot analyses (data not shown). Therefore, the transcript level of *bccnA* ^ΔAID^ mRNA was determined by quantitative RT-PCR for all three independent transformants (T1–T3) (Fig. 3). Although the wild-type and truncated gene copies are controlled by the same native promoter, the mutants revealed a dramatically reduced transcript level (about 20 to 51% of the wild-type level) suggesting a low transcript stability of the truncated gene compared to the wild-type *bccnA* gene. The reason for low transcript stability might be due to the missing terminator of the truncated *bccnA* ^ΔAID^ gene copy. Therefore, a new vector containing the *bccnA* ^ΔAID^ allele with the native terminator was transformed into the *B. cinerea* wild-type. In several transformants, the wild-type *bccnA* gene was replaced by the *bccnA* ^ΔAID^ gene. However, these mutants showed a wild-type like phenotype, normal growth, conidiation and sclerotia formation indicating that the encoded BcCnA^ΔAID^ protein does not act as a dominant active calcineurin.

### Identification of the *B. cinerea* Calcipressin Ortholog

To further study the regulation of calcineurin activity we analyzed the role of calcipressin, which has been shown to have both activating and inhibiting function on calcineurin activity in *S. cerevisiae* [41].

We identified BC1G_07084.1 (CCD44521) as a potential ortholog of the *S. cerevisiae* regulator of calcineurin RCN1, based on highest similarity to the yeast protein. The predicted open reading frame of *bcrcn1* contains no introns and encodes 255 aa. Standard protein blast revealed an overall amino acid identity of 92% (E value 3e-152) to the *S. sclerotiorum* ortholog, 48% (6e-55) to *A. fumigatus* CbpA, 34% (2e-12) to *C. neoformans* Cbp1 and 27% (8e-08) to *S. cerevisiae* RCN1. As all important regions, such as the N-terminal domain, the SP-linker domain for phosphorylation and the C-terminal domain, were present with a significant level of sequence conservation in the potential ortholog, we assumed that *bc1g_07084.1* encodes the calcipressin ortholog BcRcn1 (data not shown).

### BcRcn1 is Important for Vegetative Growth, but not for Sporulation or Sclerotia Formation

To analyze the function of Rcn1 in *B. cinerea*, *bcrcn1* was disrupted. The *bcrcn1* replacement fragment (Fig. S2B) was transformed into protoplasts of *B. cinerea* B05.10 resulting in three independent Δ*bcrcn1* mutants. For verification that the phenotype of Δ*bcrcn1* was due to the gene deletion, Δ*bcrcn1* mutant T3 was transformed with a construct comprising the entire *bcrcn1* wild-type allele and its 5′- and 3′-non-coding regions. Three independent complementation transformants (*bcrcn1*com) were obtained for further studies.

Radial growth of Δ*bcrcn1* and *bcrcn1*com strains was analyzed on different solid media. As all deletion mutants on one hand, and all complementation strains on the other hand showed a similar phenotype, only one transformant per mutation was used for subsequent studies. Deletion of *bcrcn1* resulted in formation of small, compact colonies (Fig. 2A) and markedly decreased growth rates. The growth defect of Δ*bcrcn1* mutants was completely restored in the complementation mutants (data not shown). The *bcrcn1* deletion strains were not impaired in conidiation, conidia germination or sclerotia formation.

### High Osmolarity Improves the Growth of Δ*bcrcn1* and Δ*bccnA* Mutants

**Figure 4:**
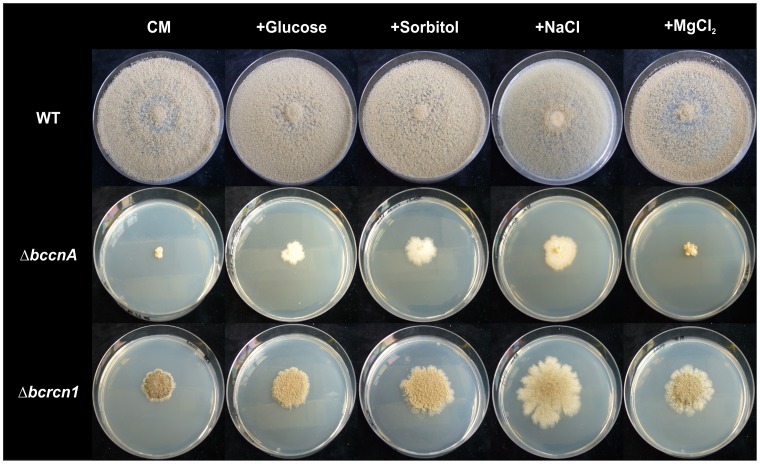
Phenotypical comparison of calcium signaling mutants. Pictures were taken after incubation of growing strains for 2 weeks on the indicated media. Glucose, sorbitol and NaCl were added with a molarity of 1 M to the basic CM medium, MgCl_2_ with a concentration of 67 mM.

On media with high-osmolarity caused by addition of salts (NaCl) or sugars (sorbitol, glucose, sucrose), growth rates of Δ*bcrcn1* mutants were slightly improved. The most striking increase in growth rate was observed for Δ*bcrcn1* mutants on CM medium supplemented with NaCl (Fig. 4). The growth rates of Δ*bccnA* mutants were increased by addition of 1 M sorbitol, glucose or NaCl to solid complete medium. In contrast to the deletion mutant of the Ca^2+^/CN-dependent transcription factor BcCrz1 whose growth was almost fully restored by Mg^2+^ ions [34], the growth rate of Δ*bccnA* mutant was not significantly improved by MgCl_2_ supplementation (Fig. 4).

### BcRcn1 is Essential for Full Virulence

In contrast to *bccnA* mutants which were completely avirulent (Fig. 1), Δ*bcrcn1* mutants were able to penetrate, but spreading lesion formation was retarded. Complemented transformants (*bcrcn1*com) were fully pathogenic (Fig. 2B). However, using young non-sporulating mycelia of the Δ*bcrcn1* strain for inoculation, no disease symptoms developed, and conidiophores and conidia formed only on the agar plugs (data not shown).

### BcRcn1 is Localized Basically in the Cytoplasm, but Accumulates at the Nuclei

**Figure 5:**
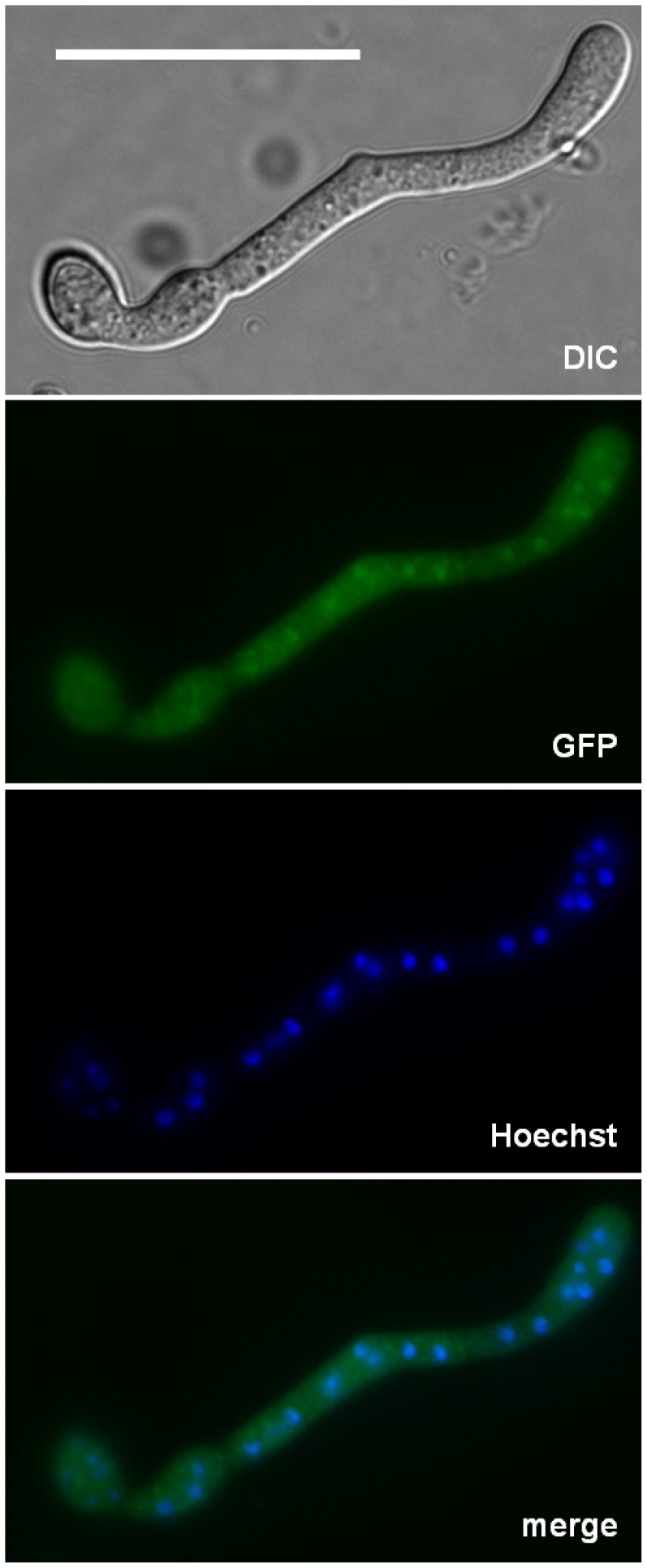
Localization of GFP-BcRcn1 in *B. cinerea*. The strain Δ*bcrcn1* was transformed with a *gfp*-*bcrcn1* fusion construct under the control of the constitutive *oliC*-promoter. Fluorescent protein was demonstrated to be homogenously distributed at a basal level in the cytoplasm but accumulated around or in the nuclei. Nuclei were visualized by using the fluorescent dye Hoechst 33342 (for details see Materials and Methods). Scale bar: 20 µm.

To study the subcellular localization of BcRcn1, *bcrcn1* was fused to codon-optimized *gfp* [48]. The Δ*bcrcn1* mutant transformed with the *gfp*-*bcrcn1* fusion construct showed complete restoration of growth and pathogenicity, demonstrating full functionality of the fusion protein (data not shown). Fluorescence microscopy of this GFP-marked strain showed both, a homogenous distribution of a basic level of BcRcn1 in the cytoplasm, but also a stronger fluorescence around or in the nuclei, when monitored by simultaneous staining of the nuclei with Hoechst 33342 (Fig. 5). This localization pattern of GFP-BcRcn1 was shown under standard cultivation conditions (GB5 medium), but also under stress conditions, e.g. upon addition of 20 mM CaCl_2_ or at alkaline pH (data not shown).

### Motif Characterization of BcRcn1

**Figure 6:**
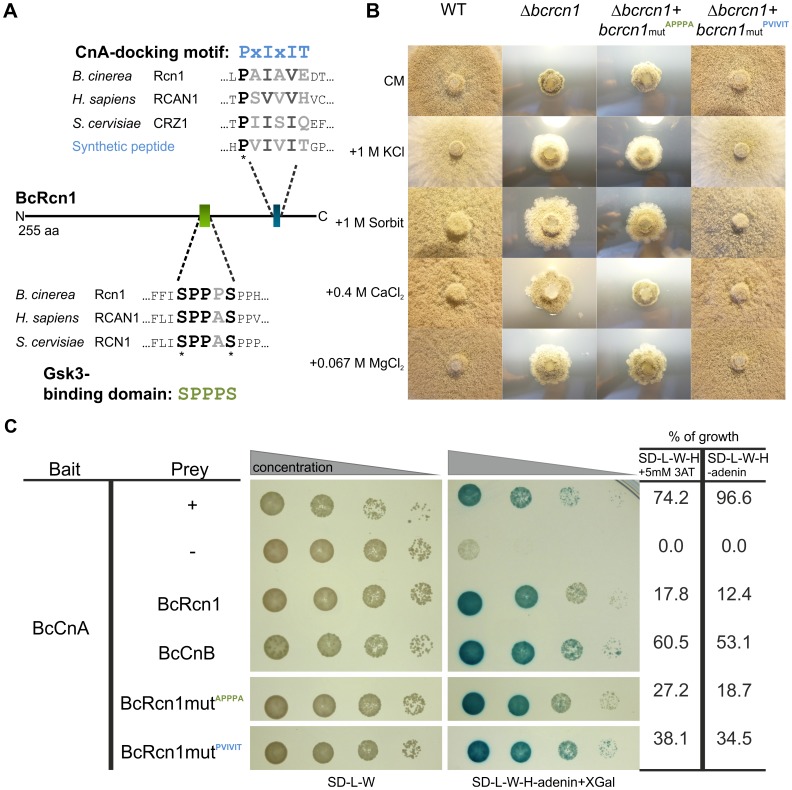
Characterization of calcipressin and calcineurin A interaction in *B. cinerea*. A: Alignments of the predicted CnA-docking motif and the putative Gsk3-binding domain in the BcRcn1 background (BcRcn1 protein: 255 aa). Mammalian and yeast as well as the synthetic peptide sequences derive from [45]. Black letters/stars: highly conserved residues; dark grayish letters: amino acids with strongly similar properties; light gray letters: weakly similar properties. B: Phenotype of mutated strains: growth analyses with wild-type B05.10 (WT), Δ*bcrcn1*, Δ*bcrcn1* + *bcrcn1*mut^APPPA^, Δ*bcrcn1* + *bcrcn1*mut^PVIVIT^ after 1 week on described media. Inoculation was made with agar plugs of equal size (5 mm). C: Yeast strain NMY51 was transformed with the bait vector containing the *bccnA* gene and different prey vectors: + control: pAI-Alg5, - control: pPR3-N, BcRcn1/BcCnB/BcRcn1mut^APPPA^/BcRcn1mut^PVIVIT^: pPR3-N_BcRcn1/_BcCnB/_BcRcn1mut^APPPA^/_BcRcn1mut^PVIVIT^. Strains were incubated in different concentrations (non-diluted, 1∶10, 1∶100, 1∶1000-diluted) on SD-L-W (selection for vectors) and on SD-L-W-H-adenin + X-Gal-plates (interaction of tested proteins). Percentage of growth was calculated by counting of colonies in comparison to number of colonies on SD-L-W. The test was performed three times.

Calcipressins have two phosphorylation sites (serine residues). In *S. cerevisiae*, their phosphorylation status decides on whether they have an inhibiting or activating function on CN [12], [41]. Potential phosphorylation sites were identified in the amino acid sequence of BcRcn1 by a multiple sequence alignment (Fig. 6A). The most conserved motif, the so-called SP-motif FxISPPxSPP, is displayed in the central span of about 20 aa. As previously described by Hilioti and Cunningham [41], the SP-linker region containing these two conserved serine-proline repeats is essential for phosphorylation of Rcn1 by the kinase Gsk3. In yeast and mammals, the protein kinase Gsk3 phosphorylates Rcn1 at the first serine residue in this domain after priming phosphorylation has taken place at the second serine residue. Beside the potential phosphorylation motif, a second conserved motif was found which is similar to the CN-specific PxIxIT docking site [42], [45], [47].

To study the function of these conserved motifs in more detail, we performed site-directed mutagenesis of *bcrcn1* and expressed the mutated gene copies individually in the Δ*bcrcn1* background (Fig. 6B). First of all, we mutated the SPPPS motif to an APPPA motif which can probably no longer be phosphorylated. Furthermore, to show whether BcRcn1 and BcCnA interact and whether the suggested interaction site is functional, we generated an altered PxIxIT CN-docking site in BcRcn1. By this procedure, the motif was changed from PAIAVE to PVIVIT, a motif which was shown to have the highest affinity to CNA when different synthetic peptides were tested as substrates [46].

Vectors carrying the two mutated *bcrcn1* copies under control of the native *bcrcn1* promoter were transformed into the Δ*bcrcn1* strain. Interestingly, transformants carrying *rcn1*mut^APPPA^ with the mutated phosphorylation sites showed exactly the same phenotype as the *bcrcn1* deletion mutant or the wild-type when treated with the CN-inhibitors CsA or FK506 [34]. These data correlate with the suggestion that non-phosphorylated BcRcn1 functions as an inhibitor of CN activity.

In contrast, strains expressing the *bcrcn1* allele with the proposed higher-affinity motif PVIVIT revealed a wild-type-like phenotype. A higher affinity of BcRcn1 to BcCnA seems to have no effect on vegetative growth or pathogenicity.

### BcRcn1 and BcCnB Interact with BcCnA in Yeast

To experimentally prove whether the wild-type and the two mutated proteins of BcRcn1 interact with BcCnA, a split-ubiquitin based yeast two-hybrid approach was performed. Therefore, a bait-vector with *bccnA* encoding the catalytic subunit of CN and several prey vectors containing the *bcrcn1* wild-type gene, one of the *bcrcn1* mutant gene copies or the *bccnB* gene encoding the regulatory subunit of CN were cloned and transformed into yeast for interaction studies. As expected, BcCnA clearly interacts with its putative regulator BcRcn1 and the regulatory subunit BcCnB (Fig. 6C). The mutated proteins (BcRcn1mut^APPPA^, BcRcn1mut^PVIVIT^) also showed an interaction with BcCnA. BcRcn1mut^PVIVIT^ revealed even significantly higher interaction rates with BcCnA than the wild-type protein (comparable to those of BcCnB) demonstrating that the mutated PVIVIT motif has indeed a higher affinity to CNA.

### BcRcn1 Regulates BcCnA-dependent Gene Expression

**Figure 7:**
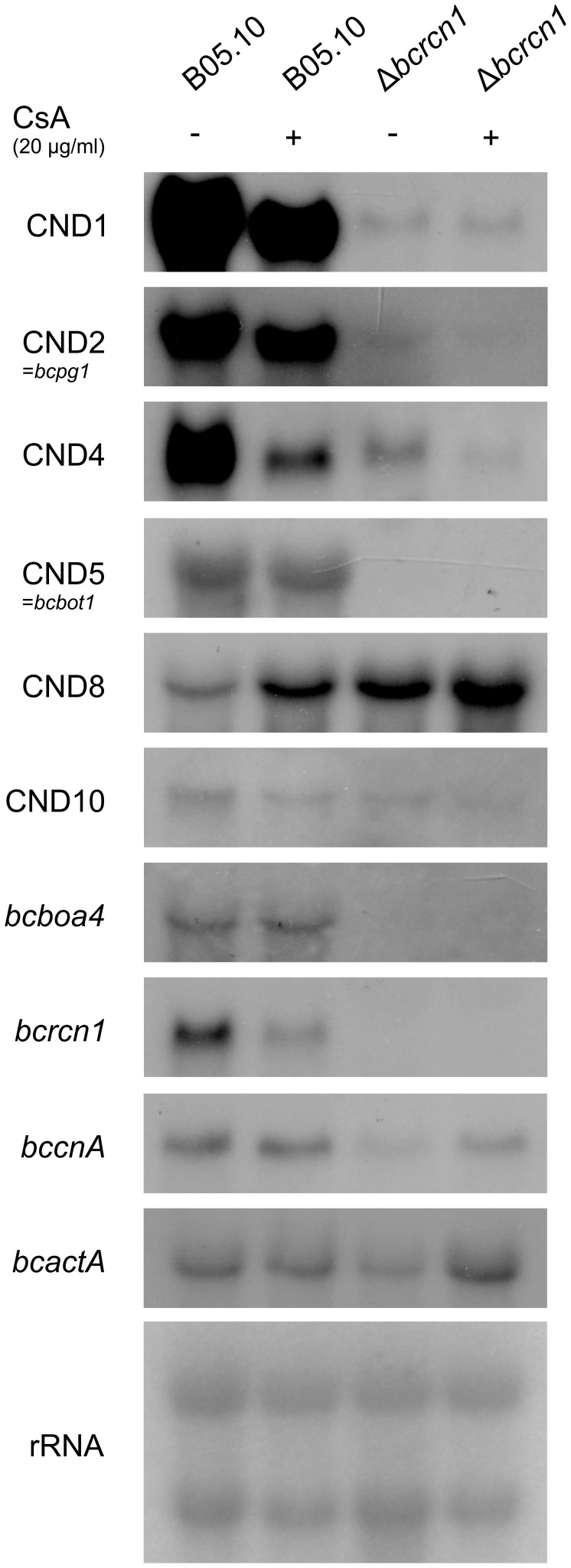
Expression studies in the wild-type and the Δ*bcrcn1* mutant strain. The wild-type B05.10 and the Δ*bcrcn1* mutant were grown for 72 h in liquid culture (SISLER medium) and then moved to fresh cultures without (−) or with (+) 20 µg/ml of the calcineurin-inhibitor cyclosporine A (CsA). The northern blot was hybridized to radioactively labeled probes of several BcCnA/BcCrz1-dependent genes (CND-genes [30], CND5 =  *bcbot1* as a botrydial biosynthesis gene, *bcboa4* as a botcinic acid biosynthesis gene), *bccnA* and *bcrcn1*. Loading controls: *bcactA* and rRNA.

To study the possible involvement of BcRcn1 in CN-dependent regulation of gene expression we studied the expression of previously identified CN target genes in both the wild-type and the Δ*bcrcn1* mutant by northern blot analyses (Fig. 7). Both strains were cultivated in liquid cultures in the presence or absence of the CN inhibitor CsA (as described by Viaud et al. [30]). All chosen CN-dependent genes showed similar expression patterns in the *bcrcn1* deletion mutant and the CN-inhibited wild-type. Only some genes were up-regulated by inhibition of CN (e.g. CND8) and in the Δ*bcrcn1* mutant. Most of the CN-dependent genes were down-regulated in the wild-type upon CsA treatment and also less expressed in the mutant. Interestingly, the expression level of *bccnA* itself was lower in the Δ*bcrcn1* mutant. For quantification of the *bccnA* transcript in the Δ*bcrcn1* background, qRT-PCR analyses were carried out confirming that the *bccnA* transcript level is significantly reduced to 42% in the Δ*bcrcn1* mutant compared to the wild-type (Fig. 3). In contrast, the *bccnA* transcript level was not significantly decreased in the Δ*bccrz1* mutant under the same conditions. However, the *bcrcn1* expression level was reduced by addition of CsA to the wild-type indicating an interdependent transcriptional regulation of BcRcn1 and BcCnA.

## Discussion

In this work, we studied the function and regulation of two components of the Ca^2+^/CN-dependent signaling pathway in the phytopathogenic fungus *B. cinerea*, calcineurin and its putative regulator, Rcn1. Previous CN inhibitory studies already demonstrated that CN is required for growth, morphogenesis, secondary metabolism and virulence in *B. cinerea* [30], [49] and that Bcg1 and CN share a set of target genes, e.g. those for botrydial and botcinic acid [35].

Here we present the successful generation of *bccnA* knock-out mutants by using osmotically stabilized selection medium. Additionally, we generated a *bccnA* ^ΔAID^ mutant expressing a truncated *bccnA* gene copy without the autoinhibitory domain. Furthermore, we identified the only member of the calcipressin family in *B. cinerea*, BcRcn1. With these mutants available, we were able to study the complex Ca^2+^/CN-dependent regulatory system by comparing the characteristic features of these mutants regarding growth, differentiation, expression pattern, and virulence with those of the wild-type.

### Calcineurin has an Impact on Growth and Development of *B. cinerea*

Previous attempts to delete the two CN subunits failed, suggesting that both genes are essential in *B. cinerea* [30]. The most obvious phenotype of the Δ*bccnA* mutants was the severe growth defect. The hyphal morphology was heavily modified and comparable to that obtained for the wild-type treated with CsA. Δ*bccnA* mutants were absolutely avirulent on wounded and unwounded bean plants. These data fit to previous penetration assays on onion epidermis which showed that reduced CN activity in wild-type conidia prevented the formation of infection structures in *B. cinerea* strain T4 [30]. Furthermore, deletion of *bccnA* or inhibition of CN by CsA probably results in inactivation of the downstream-acting transcription factor BcCrz1 that was shown to have an impact on virulence. Similarly to Δ*bccnA* mutants, *bccrz1* deletion mutants were unable to penetrate bean leaves when agar plugs were used for inoculation [34].

A characteristic feature of *bccnA* mutants is their genetic instability, for their morphology changed within weeks from dark-colored to brightly-colored colonies. All formed sectors were homokaryotic deletion mutants, indicating that genetic instability is due to a process of suppression of the mutation. The extremely low growth rate was slightly improved in media with high osmolarity indicating that the mutant is affected in cell wall stability. The other characteristic features, the altered hyphal morphology and septa formation, support the suggestion that the cell wall composition of *bccnA* mutants might differ from those in the wild-type. However, high-osmolarity conditions did not fully restore wild-type growth and morphology indicating that the mutant has additional defects. As the Δ*bcrcn1* mutants also react on these conditions with an improvement of growth we conclude that the Ca^2+^/CN-signaling pathway influences the cell wall composition in general.

In yeast cells, Mg^2+^ starvation induces the translocation of the transcription factor Crz1 from the cytoplasm into the nucleus. Mg^2+^ depletion causes enhanced cellular Ca^2+^ concentrations which activate the CN/Crz1-pathway [50]. Interestingly, in *B. cinerea*, the role of Mg^2+^ ions seems to be different. Whereas addition of external Mg^2+^ almost fully restored growth rate, conidiation and penetration, but not sclerotia formation of Δ*bccrz1* mutants [34], addition of external Mg^2+^ failed to overcome the growth or differentiation defects of *bccnA* mutants leading to the conclusion that Mg^2+^-dependent metabolism acts downstream of CN.

Surprisingly, the deletion of the AID caused a reduced transcript level of *bccnA* that was confirmed by qRT-PCR. The *bccnA* ^ΔAID^ vector was cloned without the 3′-UTR to avoid loss of the resistance cassette by homologous recombination at the terminator region. Transcript instability due to the missing terminator might be the reason for the unexpected decrease of the transcript level of *bccnA* in the *bccnA* ^ΔAID^ mutant. Subsequently, the mutant shows a phenotype resembling that of the Δ*bcrcn1* mutant and the CsA-inhibited wild-type. On the other hand, the less severe phenotype of the *bccnA* ^ΔAID^ mutant compared to the Δ*bccnA* mutant must be due to a partially functional BcCnA^ΔAID^ protein as the *bccnA* ^ΔAID^ allele is the only available *bccnA* gene copy in these mutants. In addition, transformants carrying the *bccnA* ^ΔAID^ allele with the native terminator showed a wild-type-like phenotype. In conclusion, deletion of the AID did not result in the expected dominantly active calcineurin.

It might be possible to generate constitutively active forms of CN by deleting both, the calmodulin binding domain and the AID, as shown by O’Keefe et al. [51]. The regulatory role of the C-terminal domains of CNA in mammals was explored by Wang et al. [52], [53]. They showed that not only the AID, but two distinct segments are responsible for inhibiting the enzymatic activity by binding to the catalytic site and to the CNB binding helix. Future experiments will show if the deletion of both domains as well as site-directed mutagenesis on specific residues result in a dominantly active CnA in *B. cinerea*.

### BcRcn1 is Accumulated Around the Nuclei

Little is known about the subcellular localization of components of the Ca^2+^/CN-signaling pathway, such as the CN subunits, or the potential CN regulator Rcn1, in filamentous fungi. For the transcription factor Crz1 it has been shown that it is translocated to the nucleus upon its dephosphorylation by CNA [26], [34]. *A. fumigatus* was the first filamentous fungus in which the catalytic subunit of calcineurin was shown to localize at the hyphal septum where it complexes with CnaB for normal CN function [54].

In this work, we investigated localization of BcRcn1 by expressing a GFP fusion construct. The GFP-BcRcn1 fusion protein was distributed homogenously in the cytoplasm, as described for *A. nidulans* [39]. However, partial accumulation of BcRcn1 was also observed around or in the nuclei under all tested conditions. This is the first indication that BcRcn1 may modulate CN activity in the cytoplasm in near proximity to or in the nucleus. Its modulation can take place either by specific regulation of CN activity to mediate the Ca^2+^ signaling response, or by its targeting to the nucleus for further facilitation of substrate recognition. Future localization studies of BcCnA would give further insight into the complex interaction network of these diverse signaling components.

### BcRcn1 is a Regulator of Calcineurin Activity

To show whether CN activity in *B. cinerea* is regulated in a similar way as in yeast and mammals, we studied the role of calcipressin, BcRcn1, on Ca^2+^/CN-dependent signaling. Deletion of *bcrcn1* resulted in reduced hyphal growth as previously reported for the corresponding mutant in *S. cerevisiae* [12] and the human pathogen *A. fumigatus* [40].

The Δ*bcrcn1* phenotype showed a strong similarity to the wild-type treated with CN inhibitors suggesting that BcRcn1 acts as a positive regulator of CN activity and the Ca^2+^/CN-dependent pathway in *B. cinerea*. In contrast, Rcn1 functions as an inhibitor as well as an activator of CN signaling in yeast [12], [41].

### Expression Studies Confirm the Activating Role of BcRcn1

To confirm that BcRcn1 is an activator of CN activity in *B. cinerea*, we studied the expression of CN-dependent genes in the wild-type and *bcrcn1* mutants, treated or non-treated with CsA. Deletion of *bcrcn1* resulted in the same effect as inhibition of CN in the wild-type. Among the CN target genes were also those involved in secondary metabolism, like botrydial biosynthesis genes, e.g. *bcbot1* (CND5) [30], [55]. While the inhibition of CN by CsA did not affect the level of *bccnA* transcript, the latter was significantly decreased in the Δ*bcrcn1* mutant independently of the presence of CsA. On the other hand, the *bcrcn1* transcript itself was differentially expressed in a CN-dependent manner, as a lower transcript level was observed after treatment with CsA. Furthermore, we found a putative CDRE (calcineurin-dependent response element) in the promoter region of *bcrcn1* at position 746 bp upstream of the start codon. The identified motif GTGGCTGGG fits in the model element G[T/G]GGC[T/A]G[T/G]G predicted as binding motif for Crz1 in *A. nidulans* [56]. These results indicate an interconnection of BcCnA and BcRcn1 at transcriptional level via binding of BcCrz1 to the promoter of *bcrcn1* similarly to *A. nidulans*. Dependent on the environment, active BcCnA enhances *bcrcn1* expression via BcCrz1. On the other hand, BcRcn1 positively regulates *bccnA* expression.

In *A. fumigatus*, CbpA is probably also involved in the transcriptional regulation of CN, while Cbp1 in *C. neoformans* did not significantly affect the transcriptional level of *cna1* [40], [57]. In *S. cerevisiae*, neither the deletion nor the overexpression of *rcn1* affected the expression of an epitope-tagged Cna1-Myc fusion under non-signaling conditions. However, under high-calcium conditions, the expression of the *cna1* fusion consistently declined to lower expression levels in Δ*rcn1* mutants [12]. In contrast, the co-regulation of all CND genes in *B. cinerea* indicated an exclusively activating function of BcRcn1 on the CN signaling cascade.

### Modulatory Effect of BcRcn1 through Direct Interaction with BcCnA

In this study we showed that the catalytic subunit BcCnA not only physically interacts with its regulatory subunit, BcCnB, but also with BcRcn1 further indicating the role of calcipressin as regulator of calcineurin. To study the role of putative functional domains of BcRcn1, different mutations were introduced into the *bcrcn1* gene. Mutation of both putative phosphorylation sites in BcRcn1 did not result in any change of the interaction rate with BcCnA, but in a phenotype resembling the knock-out of *bcrcn1* or the CsA-treated wild-type [34]. Apparently, the activating effect of BcRcn1 on the Ca^2+^ signaling cascade is missing in this mutant, confirming our model of BcRcn1 as an activator of CN activity. In yeast, an activating function of Rcn1 was suggested for the double-phosphorylated form, whereas dephosphorylation causes inhibition of CN [37]. Mutations of only one of the two phosphorylation sites could help to elucidate the role of the phosphorylation status in modulating CN activity.

In addition, we were able to demonstrate that the PAIAVE (PxIxIT)-sequence element in BcRcn1 is a functional interaction domain with CNA. Remarkably, the replacement of the potential PxIxIT-CNA-binding motif in BcRcn1 by a motif with postulated higher affinity to CNA [46] resulted indeed in much higher interaction rates up to the same strength as for the two CN subunits, BcCnA and BcCnB. However, this much higher affinity did not result in any visible phenotype: mutants expressing the high-affinity BcRcn1 protein showed wild-type-like characteristics.

There are reports on the presence of additional interaction sites in CN substrates, such as those proposed for NFAT transcription factors in mammals, the analogue CN target of the yeast and filamentous fungal transcription factor Crz1, or on alternative mechanisms for CN-substrate interaction (reviewed in [45], [47]). There are indications that the LxVP-motif which was described as additional motif for interaction with CNA in these reports, is also present in BcRcn1 (data not shown), which may be responsible for interaction with CNA as well. Therefore further studies are needed to unravel the mechanism of regulation of CN by BcRcn1 in *B. cinerea*.

### Conclusions and Perspectives

Despite the high level of sequence conservation of components of signaling pathways between yeasts, filamentous fungi and higher eukaryotes, several differences have been found regarding the function of these signaling cascades in processes of life, such as vegetative growth, development, reproduction, and virulence. Nothing was known so far about mechanisms of regulation of CN in phytopathogenic fungi. In this work we demonstrated on transcriptional and protein levels that the putative CN regulator BcRcn1 is an activator of CN activity in *B. cinerea* under all tested conditions.

This is the first report of calcipressin acting as a positive regulator of CN and CN target genes, such as those involved in secondary metabolism. Future studies will show under which conditions the Ca^2+^/CN-dependent signaling cascade is activated in *B. cinerea* and whether BcRcn1 has an inhibiting function on CN as well under those conditions.

Mutants containing a GFP-BcCrz1 fusion protein will be used for studying factors affecting nuclear translocation of this transcription factor.

## Methods

### Strains and Culture Conditions

*B. cinerea* Pers.:Fr. [teleomorph *Botryotinia fuckeliana* (de Bary) Whetz] Strain B05.10 is a putative haploid strain obtained after benomyl treatment of an isolate from *Vitis* [58] and is used as a host strain for gene replacement experiments and as wild-type control. Wild-type and mutant strains were grown on several complex media: potato dextrose agar (Sigma-Aldrich Chemie, Steinheim, Germany) was supplemented with 10% homogenized leaves of French bean (*Phaseolus vulgaris*) (PDAB). Synthetic complete medium (CM) was made according to Pontecorvo et al. [59]. As minimal medium, modified Czapek-Dox (CD) medium (2% sucrose, 0.1% KH_2_PO_4_, 0.3% NaNO_3_, 0.05% KCl, 0.05% MgSO_4_ ×7 H_2_O, 0.002% FeSO_4_ ×7 H_2_O, pH 5.0) or GB5 (0.033% Gamborg’s B5 [Duchefa Biochemie BV, Haarlem, The Netherlands], 2% glucose) was used. For conidiation, the strains were incubated at 20°C under light (12 h light/12 h darkness) conditions; for sclerotia formation, they were incubated at 20°C in continuous darkness. For DNA and RNA mini-preparations, mycelium was grown for 3 to 4 days at 20°C on CM agar (+/− supplements) with a cellulose acetate (Cellophane) overlay or in liquid culture (SISLER medium). For northern blot and qRT-PCR analyses strains were treated as described by Viaud et al. [30]. Plate assays were performed using CM agar with or without the mentioned supplements as indicated.

Yeast cells were grown either in complete YP(A)D medium consisting of 2% glucose, 2% peptone, (0.004% adenine sulfate) and 1% yeast extract or in minimal SD medium containing 2% glucose, 0.67% yeast nitrogen base, and the amino acids required by the strains. Solid media contained 2% agar. *S*. *cerevisiae* strains were transformed by the lithium acetate procedure as described previously [60]. For maintenance of plasmids, yeast transformants were pre-cultured in selective media.

*Escherichia coli* strains TOP10F’ (Invitrogen, Groningen, Netherlands) and *E. coli* XL1-Blue (Agilent Technologies) were used as hosts for plasmid construction, site-directed mutagenesis and propagation.

### Germination Assays and Microscopic Analyses

Fluorescence microscopy was carried out after incubation of germinating conidia for 16–24 hours post inoculation (hpi) in liquid GB5 medium supplemented with 2% glucose and 0.132 g/l (NH_4_)_2_HPO_4_. To study hyphal morphology, the *B*. *cinerea* strains were grown in liquid CM supplemented with cyclosporine A or CaCl_2_ as indicated. After incubation for 24–48 hpi at 20°C, the colonies were incubated for 5 min in 1% (wt/vol) Calcofluor White solution. For detection of nuclei, nucleic acids were stained using the fluorescent dye Hoechst 33342; 10 µl freshly prepared Hoechst solution, prepared according to Kangatharalingam et al. [61], were added to the germinated conidia prior to microscopy. Fluorescence and light microscopy was performed with an Axio Imager.M2 microscope (Carl Zeiss MicroImaging GmbH, Jena, Germany). Differential interference contrast (DIC) microscopy was used for bright field images. Staining of hyphae by Hoechst 33342 and Calcofluor White was examined using the filter set 49 DAPI shift free (excitation G 365, beam splitter FT 395, emission BP 445/50) and GFP fluorescence with filter set 38 (excitation BP 470/40, beam splitter FT 495, emission BP 525/50). Images were captured with an AxioCam MRm camera and analyzed using the Axiovision Rel 4.5 software package (both Carl Zeiss MicroImaging GmbH, Jena, Germany).

### Pathogenicity Assays on Bean Plants

Infection assays were performed with conidia from 7 to 10-day-old PDAB agar cultures as described previously [32]. In addition, non-sporulating agar plugs taken from 3-day-old CM agar cultures were used to inoculate primary leaves of *Phaseolus vulgaris* on the adaxial surface. The infected plants were incubated in a plastic propagator box at 20°C under natural illumination. Disease symptoms were scored until 10 days after inoculation.

### Standard Molecular Methods

Fungal genomic DNA was isolated as described previously [62]. Plasmid DNA was isolated using a plasmid DNA preparation kit (Genomed, Bad Oeynhausen, Germany). For Southern blot analyses, the fungal DNA was transferred to Hybond N^+^ filters (Amersham Biosciences, Freiburg, Germany) after digestion with restriction enzymes and size separation on a 1% agarose gel according to the method of Sambrook et al. [63]. Hybridization was carried out in 6x SSC (1x SSC is 0.15 M NaCl plus 0.015 M sodium citrate), 5x Denhardt’s solution, 0.1% SDS, and 50 mM phosphate buffer, pH 6.6, at 65°C in the presence of a random-primed [α-^32^P]dCTP-labeled probe. The membranes were washed once (2x SSPE [1x SSPE is 0.18 M NaCl, 10 mM NaH_2_PO_4_, and 1 mM EDTA {pH 7.7}], 0.1% SDS) before being exposed to autoradiographic film. Total RNA was isolated from mycelial samples using the Trizol procedure (Invitrogen, Groningen, The Netherlands). Samples (25 µg) of total RNA were transferred to Hybond N^+^ membranes after electrophoresis on a 1% agarose gel containing formaldehyde, according to the method of Sambrook et al. [63]. Blot hybridizations were done in 0.6 M NaCl, 0.16 M Na_2_HPO_4_, 0.06 M EDTA, 1% *N*-lauroylsarcosine (Sigma-Aldrich, St. Louis, MO), 10% dextran sulfate (Eppendorf AG, Hamburg, Germany), 0.01% salmon sperm DNA, pH 6.2, as described for Southern blots; 1 µg of total RNA was taken for cDNA synthesis using the oligo(dT)12–18 primer and SuperScript II reverse transcriptase (Invitrogen, Groningen, The Netherlands) according to the manufacturer’s instructions. PCR mixtures contained 25 ng DNA, 5 pmol of each primer, 200 nM concentrations of deoxynucleotide triphosphates, and 1 unit of BioThermDNA polymerase (GeneCraft GmbH, Lüdinghausen, Germany). The reactions started with 4 min at 94°C, followed by 35 cycles of 1 min at 94°C, 1 min at 56 to 65°C, and 1 min at 70°C, and a final 10 min at 70°C. PCR products were cloned into pCR®2.1-TOPO® (Invitrogen, Groningen, The Netherlands). For sequence analyses, Lasergene v6 software (DNAStar, Madison, WI) was used. Quantitative real-time PCR was carried out using a one-tenth dilution of the cDNA template in a MyiQ2 Two-Color Real-Time PCR Detection system (Bio-Rad, Hercules, CA, U.S.A) with the Bio-Rad iQ SYBR Green supermix. The genes encoding actin A, glyceraldehyde-3-phosphate dehydrogenase and tubulin showed the same expression pattern in the wild type strain, as well as in the mutant strains used in this work. All three genes were used to normalize the cDNA amount in each sample using the primers 1/2 (for actin A), 3/4 (glyceraldehyde-3-phosphate dehydrogenase) and 5/6 (tubulin). All primers used in this study are listed in the Table S1. To study the expression of *bccnA* in the *bccnA* ^ΔAID^ mutants, primers 7/8 were used. The annealing temperature in every PCR was in a range from 58°C to 62°C, while the time extension was always 20 s. To avoid genomic DNA amplification, RNA samples were treated with DNAseI (Promega) and every pair of primers was designed in such way that one of them hybridized to an exon-exon split. Therefore, for each gene, the PCR efficiency was between 90 and 110%. The relative expression of *bccnA* was calculated following the ΔΔCt (cycle threshold) method, from the mean of two different determinants of Ct values.

### Vector Cloning

For construction of the vector pΔ*bccnA-hyg^R^*, the plasmid pOliHP [64], carrying the *E. coli* hygromycin B phosphotransferase gene *hph* under control of the *A. nidulans oliC* promoter, was used as basal vector (Fig. S2A). The gene flanks were amplified by PCR with primers, derived from the genomic sequence of *B. cinerea* B05.10 (ABN58724), containing artificial restriction sites for further cloning. A 1.4-kb PCR fragment was amplified from the *bccnA* 5′-region using the primers 9&10, and a 0.6-kb fragment of the 3′-untranslated region was generated as second flank using primers 11&12. Both PCR products were cloned into pCR®2.1-TOPO® (Invitrogen, Groningen, The Netherlands), isolated with KpnI/SalI and HindIII/EcoRI, respectively, and then cloned into the corresponding restriction sites of pOliHP, creating pΔ*bccnA-hyg^R^*. Prior to transformation, the whole replacement cassette was isolated by restriction with KpnI and EcoRI. Hygromycin B-resistant transformants were analyzed by PCR for homologous integration using the primers 13&14 and 15&16. Putative knock-out mutants were purified by several rounds of single spore isolation and screened by PCR for the absence of the *bccnA* wild-type allele using the primers 17&18 (Fig. S2A). Southern blot analyses of these transformants was not feasible as mutants were so slow growing that genomic DNA was obtained from two to three week-old liquid cultures. The by this procedure gained DNA was so instable and difficult to obtain that just PCR was performable, but the quality was not good enough to perform restrictions for Southern blot analyses.

For expression of a truncated version of *bccnA*, pΔ*bccnA-hyg^R^* was used as basis for cloning and for mediation of homologous integration of the truncated gene at the *bccnA* locus. The gene including the promoter sequence was amplified by PCR in two steps using primers which contain restriction sites if necessary for further cloning. A 1.6-kb fragment including 1 kb of the 5′ noncoding region and 0.6 kb of the *bccnA* open reading frame by using the primers 19&20 was amplified (Fig. S2A). The second part of the gene lacking the last 250 bp was amplified by using the primers 21&22, yielding a PCR fragment with a size of 1.0 kb. Both PCR products were cloned into pCR®2.1-TOPO®, separately, then sequenced and finally isolated with KpnI/BglII and BglII/SalI, respectively. Both fragments were cloned into the KpnI and SalI restriction sites of pΔ*bccnA-hyg^R^*, creating p*bccnA* ^ΔAID^. By this approach the former 5′-flank of *bccnA* was replaced by the truncated version of *bccnA* which functions as new 5′-flank for homologous integration at the *bccnA* locus, and therefore the wild-type allele is replaced by the truncated version. For transformation of *B. cinerea* B05.10 wild-type, p*bccnA* ^ΔAID^ was linearized with SmaI, cutting behind the *bccnA*-3′-flank. The hygromycin resistant transformants were analyzed by PCR for homologous integration using the primers 13&14 (5′-flank) and 15&16 (3′-flank). Putative *bccnA* ^ΔAID^ mutants were purified by single spore isolation and screened by PCR for the absence of the *bccnA* wild-type allele using the primers 17&18. For Southern blot analysis, the genomic DNA of *bccnA* ^ΔAID^ mutant and wild-type strains was digested with KpnI, and hybridized with the *bccnA*-3′-flank after electrophoresis and blotting. The replacement of the wild-type allele by the truncated form (*bccnA* ^ΔAID^) leads to a hybridizing fragment of approx. 4 kb, in comparison to the wild-type fragment which has a size of 2 kb. The shift is due to the insertion of the hygromycin resistance cassette between *bccnA* and the 3′-non-coding region of *bccnA* (Fig. S2A).

The knock-out construct for deletion of *bcrcn1* was obtained using the homologous recombination system in yeast [65]. About 1 kb of the 5′- and the 3′-flanking regions of *bcrcn1* (CCD44521) were amplified from genomic DNA using the primer pairs 23&24 and 25&26, respectively (Fig. S2B). These primers are composed of one part for binding to the genomic DNA and the other part with homologous regions to the resistance cassette or the shuttle vector pRS426, respectively [66]. The hygromycin resistance cassette of pOliHP [64] containing the *hph* gene of *E. coli* under control of the *oliC* promoter of *A. nidulans* was amplified by PCR using primers 27&28. After restriction of pRS426 using EcoRI/XhoI transformation into yeast strain FY834 [67] with all three PCR fragments together was carried out. Positive transformants were selected on SD-uracil plates. Total DNA from uracil-prototrophic yeast colonies was isolated with the GeneJET™ Plasmid Miniprep Kit (Fermentas), using glass beads for lysation of yeast cells in the first step. The complete recombined replacement fragment for deletion of *bcrcn1* was re-amplified from yeast DNA by PCR using the primers 15&18 and taken for transformation of *B. cinerea* B05.10. Successful deletion was verified via diagnostic PCR using primers 29&30 for integration of the *hph^R^* cassette at the 5′ region, and 14&31 for the 3′ region. Absence of a wild-type copy of *bcrcn1* was proved by PCR with primers 32&33. The complete absence of any additional ectopic integration was confirmed by Southern blot analysis after digestion of genomic DNA with BamHI and hybridization with the *bcrcn1*–5′-flank. The replacement of the wild-type *bcrcn1* allele by the *hph^R^* cassette leads to a hybridizing fragment of approx. 1.4 kb, in comparison to the wild-type fragment which has a size of more than 8 kb (Fig. S2B).

For complementing the Δ*bcrcn1* mutant, a fragment containing about 1 kb of the native *bcrcn1* promoter, the complete open reading frame, and about 500 bp of the *bcrcn1* terminator was amplified by PCR using primers 34&35. The PCR product was cloned into pCR®2.1-TOPO®, resulting in pTOPO_*rcn1*com, sequenced and finally isolated with SpeI/XhoI. The fragment was cloned into pΔ*bcniaD-nat^R^*, containing the nourseothricin resistance cassette, with *nat1* from *Streptomyces noursei* under the control of the *A. nidulans trpC* promoter, creating p*bcniaD_nat* ^R^_*rcn1*com. This plasmid also contains the non-coding 5′ and 3′ flanking regions of *bcniaD*, encoding the nitrate reductase, to mediate homologous integration of the complementation construct at the *bcniaD* locus [35]. Prior to transformation the plasmid was linearized by restriction with SacI/PvuI. The resulting fragment of 5.9 kb was used for transforming mutant Δ*bcrcn1* T3. Presence of the complete *bcrcn1* complementation construct at the *bcniaD* locus was confirmed by diagnostic PCR using primer pairs 36&37, 38&39 and 40&41 (data not shown). As deletion of *bcniaD* is not essential for the complementation, just one round of single spore isolation was done to stabilize the integration event. The wild-type copy of *bcniaD* is still present in the complementation mutants.

The *gfp*-*bcrcn1* fusion fragment was generated using the homologous recombination system in yeast [65]. The coding region of *bcrcn1* was amplified using the primers 42&43 that contain overlapping sequences homologous to the glucanase terminator and *gfp* (encoding codon-optimized GFP [48]), respectively, of the vector pNAN-OGG [68]. This vector contains flanks mediating the replacement of the gene encoding the nitrite reductase, and by this ensuring integration at a known locus. After amplification, the PCR product and the NotI-digested plasmid pNAN-OGG were co-transformed into the uracil-auxotrophic *S. cerevisiae* strain FY834. DNA of pooled yeast colonies was isolated as described above and transformed into *E. coli*. Plasmid DNA from single colonies was isolated and sequenced, resulting in the final vector pNAN-OG*^bcrcn1^*G. The whole transformation construct, containing the *bcniiA* flanks, *nat* ^R^ and the *gfp*-*bcrcn1* fusion construct flanked by the *oliC* promoter and the glucanase terminator was transformed after restriction of pNAN-OG*^bcrcn1^*G with SacII. Integration of the transformation construct into WT: B05.10 and Δ*bcrcn1* T3 was analyzed by diagnostic PCR (data not shown).

For generation of mutated versions of *bcrcn1*, pTOPO_*rcn1*com which was cloned for complementation of Δ*bcrcn1* was used as basic vector. Site-directed mutagenesis was carried out as described by the manufacturer using the QuikChange® II Site-Directed Mutagenesis Kit (Agilent Technologies). By performing the PCR using the primers 44&45 (APPPA-mutation) and 46&47 (PVIVIT-mutation), respectively, via pTOPO_*rcn1*com as template, the mutation was inserted in *bcrcn1*. As for the complementation, the constructs were then cloned into the destination vector pΔ*bcniaD*_*nat^R^* resulting in pΔ*bcniaD*_*nat^R^* _*rcn1*mut^APPPA^ and pΔ*bcniaD*_*nat^R^*_*rcn1*mut^PVIVIT^ and subsequently transformed into mutant Δ*bcrcn1* T3. Transformants were further on analyzed by diagnostic PCR (data not shown).

### Transformation of *B. cinerea*

Protoplasts were generated using a mixture of Glucanex 200G (Novozymes, Denmark), lysing enzyme (Sigma Aldrich, St Louis, MO, USA), and Yatalase (Takara Bio Ins, Shiga, Japan) added to 15–20 µg of the linearized vector, and transformed according to Siewers et al. [55]. Resistant colonies were transferred to plates containing CM agar complemented with 70 µg/ml of hygromycin B (Invitrogen, San Diego, CA, USA) or 70 µg/ml of nourseothricin (Werner-Bioagents, Jena, Germany). Single conidial isolates were obtained by spreading conidial suspensions on GB5 plates containing 70 µg/ml of hygromycin B or nourseothricin. Single, germinated conidia were transferred individually to new plates containing the selection marker. Homokaryotic transformants were generated by several rounds of single spore isolation.

### Interaction Studies

Cloning of interaction vectors, yeast transformation and quantification of interaction rates were performed as described in the manufacturer’s manual of the *DUALSystems Pairwise interaction Kit*. The pDHB1-vector was used as bait- the pPR3-N-vector as prey-basis-vector (DUALSystems Biotech). cDNA of *B. cinerea* was used as template for a PCR with primers containing additional SfiI restriction sites. The pDHB1_cnA vector was cloned using primers 48&49 for PCR, digestion of the PCR-product with SfiI and ligation into the linearised pDHB1-vector using kanamycin for *E. coli* selection. For pPR3-N_cnB and pPR3-N_rcn1 primer pairs 50&51 and 52&53 were used, respectively, followed by restriction, ligation into pPR3-N and selection of transformed *E. coli* with ampicillin resistance. Mutated versions (pPR3-N_rcn1mut^APPPA^ & pPR3-N_rcn1mut^PVIVIT^) were generated using the mutated TOPO-vectors as template. Interaction studies were performed on SD-L-W- (selection for plasmid existence), SD-L-W-H-adenin- (quantification of interaction rates) or on SD-L-W-H-adenin+X-Gal-plates (containing 80 mg/l X-Gal for visualization of interaction).

## Supporting Information

Fig. S1 **Multiple alignment and domain structure of calcineurin A protein sequences from yeast, filamentous fungi and mammals.** Shown are *B. cinerea* BcCnA (ABN58724), *S. sclerotiorum* Cna1 (ABB13418), *A. fumigatus* CnaA (XP_753703), *S. cerevisiae* calcineurin A1 (AAA34465), *R. norvegicus* (CAA40398) and *H. sapiens* calcineurin A catalytic subunit (AAB23769). The proteins contain catalytic phosphatase domains at the N-termini, the calcineurin B (CNB)-binding domain, the calmodulin (CaM)-binding domain, and the autoinhibitory (AID) domain (reviewed in [13]). Identical residues are indicated in black, similar amino acids in gray. Putative binding domains via hydrogen bonds or van der Waals’ interactions with the immunophilin/immunosuppressant complexes cyclophilin A/CsA and FKBP12/FK506 as described in [69]–[71] are indicated with colored fonts (green: CsA, blue: FK506, violet: FK506 and cylophilin A, red: CsA and FK506, orange: cylcophilin A).
(TIF)

Fig. S2 **Gene replacement of** ***bccnA,*** **deletion of the autoinhibitory domain of** ***bccnA*** **and gene replacement of** ***bcrcn1*** **using the** ***hph*** **resistance cassette.** All primers used for cloning of the replacement vectors and the diagnostic PCR analyses for homologous integration are indicated and further described in the materials and methods section. A: Physical maps of *bccnA* (wild-type, WT), the Δ*bccnA*-*hyg* ^R^ and the *bccnA* ^ΔAID^ locus. The wild-type *B. cinerea* B05.10 was transformed with the *bccnA* replacement fragment (upper panel) or the truncated *bccnA* gene (lower panel) by homologous recombination and insertion of the *hph* resistance cassette. The last 250 bp (autoinhibitory domain, AID) of *bccnA* was replaced creating the *bccnA* ^ΔAID^ mutant. Diagnostic PCRs of two different Δ*bccnA* mutants (H5 and N1) and three *bccnA* ^ΔAID^ mutants (T1, T2, T3) in comparison to the WT are depicted just as the Southern blot analysis of *bccnA* ^ΔAID^ mutants. 5′ and 3′ prove homologous integration of the *hph^R^* cassette at the *bccnA* locus. WT shows the absence of the *bccnA* wild-type allele (the reverse primer lies in the AID domain). **B:** Physical maps of the *bcrcn1* wild-type (WT) gene locus and the Δ*bcrcn1* gene locus. The *bcrcn1* gene was replaced by the *hph* ^R^ cassette in the opposite direction. Diagnostic PCR and Southern blot analysis of Δ*bcrcn1* mutants (T3, T6, T7) in comparison to the WT are depicted as well. 5′ and 3′ prove homologous integration of the *hph^R^* cassette at the *bcrcn1* locus. WT shows the absence of the *bcrcn1* wild-type allele.
(TIF)

Table S1 All primers used in this study.
(DOCX)
