# Supplementary figures and images for: The Ca2+/Calcineurin-Dependent Signaling Pathway in the Gray Mold Botrytis cinerea: The Role of Calcipressin in Modulating Calcineurin Activity
Source: PLoS One. 2012 Jul 23;7(7):e41761. doi: 10.1371/journal.pone.0041761 (PMC3402410; doi:10.1371/journal.pone.0041761)

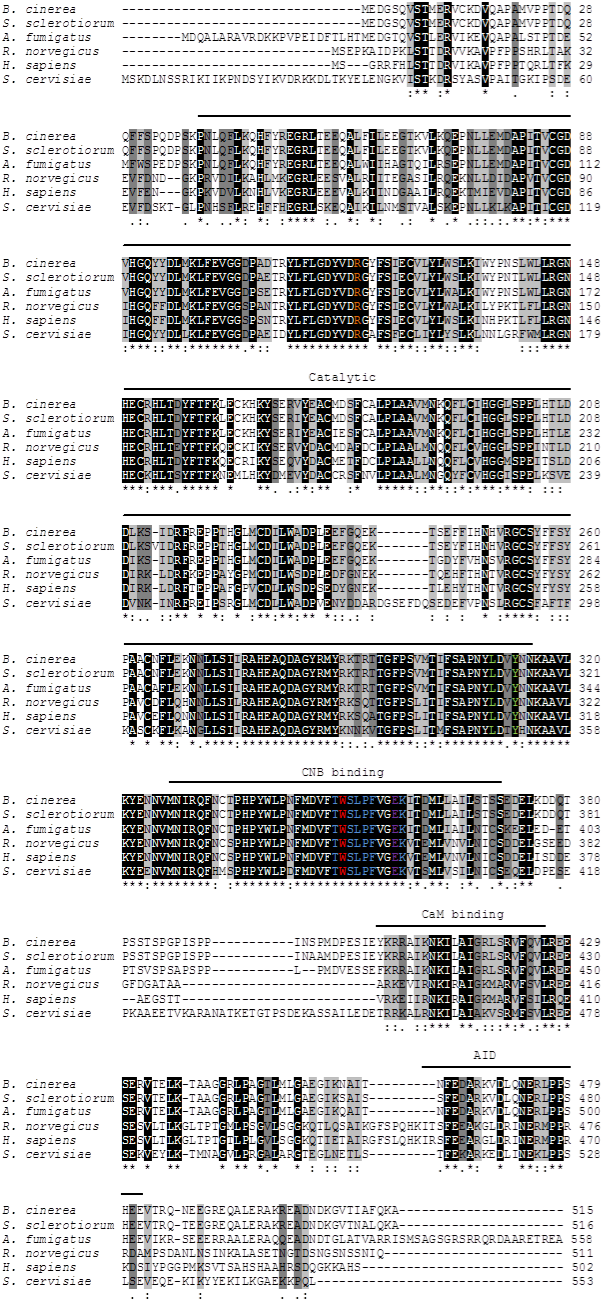

Supplement: Fig. S1 — Multiple alignment and domain structure of calcineurin A protein sequences from yeast, filamentous fungi and mammals. Shown are B. cinerea BcCnA (ABN58724), S. sclerotiorum Cna1 (ABB13418), A. fumigatus CnaA (XP_753703), S. cerevisiae calcineurin A1 (AAA34465), R. norvegicus (CAA40398) and H. sapiens calcineurin A catalytic subunit (AAB23769). The proteins contain catalytic phosphatase domains at the N-termini, the calcineurin B (CNB)-binding domain, the calmodulin (CaM)-binding domain, and the autoinhibitory (AID) domain (reviewed in [13]). Identical residues are indicated in black, similar amino acids in gray. Putative binding domains via hydrogen bonds or van der Waals’ interactions with the immunophilin/immunosuppressant complexes cyclophilin A/CsA and FKBP12/FK506 as described in [69]–[71] are indicated with colored fonts (green: CsA, blue: FK506, violet: FK506 and cylophilin A, red: CsA and FK506, orange: cylcophilin A). (TIF) [file pone.0041761.s001.tif]

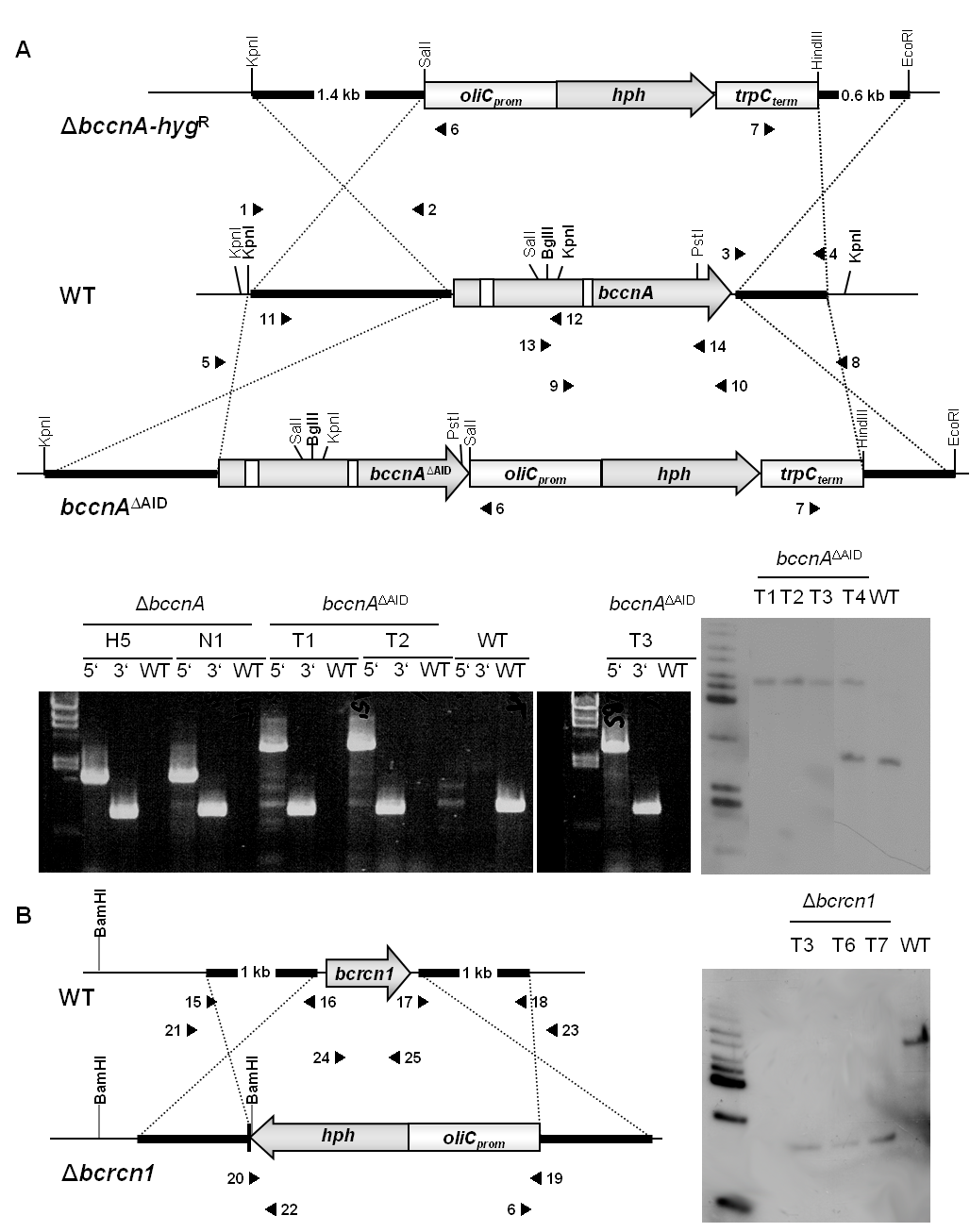

Supplement: Fig. S2 — Gene replacement of bccnA, deletion of the autoinhibitory domain of bccnA and gene replacement of bcrcn1 using the hph resistance cassette. All primers used for cloning of the replacement vectors and the diagnostic PCR analyses for homologous integration are indicated and further described in the materials and methods section. A: Physical maps of bccnA (wild-type, WT), the ΔbccnA-hyg R and the bccnA ΔAID locus. The wild-type B. cinerea B05.10 was transformed with the bccnA replacement fragment (upper panel) or the truncated bccnA gene (lower panel) by homologous recombination and insertion of the hph resistance cassette. The last 250 bp (autoinhibitory domain, AID) of bccnA was replaced creating the bccnA ΔAID mutant. Diagnostic PCRs of two different ΔbccnA mutants (H5 and N1) and three bccnA ΔAID mutants (T1, T2, T3) in comparison to the WT are depicted just as the Southern blot analysis of bccnA ΔAID mutants. 5′ and 3′ prove homologous integration of the hphR cassette at the bccnA locus. WT shows the absence of the bccnA wild-type allele (the reverse primer lies in the AID domain). B: Physical maps of the bcrcn1 wild-type (WT) gene locus and the Δbcrcn1 gene locus. The bcrcn1 gene was replaced by the hph R cassette in the opposite direction. Diagnostic PCR and Southern blot analysis of Δbcrcn1 mutants (T3, T6, T7) in comparison to the WT are depicted as well. 5′ and 3′ prove homologous integration of the hphR cassette at the bcrcn1 locus. WT shows the absence of the bcrcn1 wild-type allele. (TIF) [file pone.0041761.s002.tif]
