# Supplementary material for: The Ca2+/Calcineurin-Dependent Signaling Pathway in the Gray Mold Botrytis cinerea: The Role of Calcipressin in Modulating Calcineurin Activity
Source: PLoS One. 2012 Jul 23;7(7):e41761. doi: 10.1371/journal.pone.0041761 (PMC3402410; doi:10.1371/journal.pone.0041761)
Supplement: Table S1 — All primers used in this study. (DOCX) [file pone.0041761.s003.docx]

**Tab. S3: Primers used in this study**

| **Primer** | **Name** | **Nucleotidesequence (5’ 🡪 3’)** |
| --- | --- | --- |
| 1 | ARTPCRFW | GCTCCAAGAGCTGTTTTCCCTTC |
| 2 | ARTPCRRV | GCTTGGATTGCGCTTCATCTC |
| 3 | G3RTPCRFW | ATCGAGACTGAATATGCCGCATAC |
| 4 | G3RTPCRRV | GCCATTGACCTCCAATCCATC |
| 5 | TBRTPCRFW | ACATGCTCTGCCATTTTCCG |
| 6 | TBRTPCRRV | TTGTTAGGGATCCACTCAACGAAG |
| 7 | cna-rt-pcr-F | TGCCTTTAGCGGCTGTTATGAAC |
| 8 | cna-rt-pcr-R | CGCGGAATCGATCAATGCTC |
| 9 | bccnA‑prom‑F | GGAGAGTAGTCGCAGTAGGAAG |
| 10 | bccnA‑prom‑R | GTCGACCTTGCATCCTGTTCTCAATGCCC |
| 11 | bccnA‑term‑F | AAGCTTCCCAATCAACTAGTCATTGCTAAC |
| 12 | bccnA‑term‑R | GAATTCCACTATACCACACCCACTACACC |
| 13 | bccnA-HI-5F | GATGGTGGCAACGTTGAGGGAAGAG |
| 14 | pLOF-oliP | GGTACTGCCCCACTTAGTGGCAGCTCGCG |
| 15 | pAN-T | ACCCAGAATGCACAGGTACAC |
| 16 | bccnA-HI-3R | CTATCTACGTAGGTACCTACTTCCC |
| 17 | bccnA_gen_F | CTCACGGTCTTATGTGCGATATC |
| 18 | bccnA_gen_R | CATTATCAGCCTCCCTCTTTGC |
| 19 | bccnA-ComF1 | GGTACCGGCTCTGGCTCAGGTTCTGGGTCG |
| 20 | bccnA-ComR1 | CAGATCTTCAAAGACCAAAGATATAAC |
| 21 | bccnA-ComF2 | GTTATATCTTTGGTCTTTGAAGATCTG |
| 22 | bccnA-ComR-AID | GTCGACCAGTCTaCAGTTCGGTAACTC |
| 23 | DRcn1 5F yko | GTAACGCCAGGGTTTTCCCAGTCACGACGTAGCATCACATTACATTACATAGCCG |
| 24 | DRcn1 5R yko | ATCCACTTAACGTTACTGAAATCTCCAACGGATGTATTATGCAAGGAGGGA |
| 25 | DRcn1 3F yko | CTCCTTCAATATCATCTTCTGTCTCCGACAGTTGAGAAGCCGGGTGTGGC |
| 26 | DRcn1 3R yko | GCGGATAACAATTTCACACAGGAAACAGCATAGCAACTATCGG |
| 27 | hphF-oliC-P | GTCGGAGACAGAAGATGATATTGAAGGAGCCTGTGGAGCCGCATTCCCGATT |
| 28 | hphR-trpC-T2 | GTTGGAGATTTCAGTAACGTTAAGTGGATCGTATCTTATCGAGATCCTGAACACC |
| 29 | Bcrcn1-5F | GTAACGCCAGGGTTTTCCCAGTCACGACGCCATTGGCCACGATGTTTCG |
| 30 | pCSN44-hph-trpC-T | GGAATAGAGTAGATGCCGACCGG |
| 31 | DBcrcn1-HI-3R | GCCGCCGCATTCTAGCCCTAG |
| 32 | DRcn1-HI-WT-F | GCGCAGAGGGTCCCATAAATCG |
| 33 | DRcn1-HI-WT-R | CTTCTCCATCTCCAGCGGAGACACGC |
| 34 | Rcn1_Com_F | ATGTTGAGGCTCGGCACTT |
| 35 | Rcn1_Com_R | GCATCGCGTCACAATCTAGC |
| 36 | bcniaD_Hib_F | CGCATATCAGCATATCGAGATGTCC |
| 37 | DBcRcn13F | CTCCTTCAATATCATCTTCTGTCTCCGACGTCTGATGTGGAGACATTCG |
| 38 | bcniaD_Hia_R | GAGTACCCATCCGATGGAGTTGTTG |
| 39 | nat1-seqF1 | CGGACGGCGAGCGGCAGGCGC |
| 40 | bcniaD_WT_F | GCCACAGACTCCGCCAGATTCTAATG |
| 41 | bcniaD_WT_R | CAACCATTTCACGCTGCGACCACC |
| 42 | Bcrcn1_gfp_F | GGGAATGGATGAACTTTACAAAATGTCATCTCCAACTTCCCC |
| 43 | bcrcn1_gfp_R | CATACATCTTATCTACATACGCTAAAACTCCATCAACTCGA |
| 44 | rcn1-APPPA-forw | GAAAACTATTTTTCATTGCCCCCCCTCCCGCTCCTC |
| 45 | rcn1-APPPA-rev | GAGGAGCGGGAGGGGGGGCAATGAAAAATAGTTTTC |
| 46 | rcn1-PVIVIT-forw | GACACAGTCCTAATTTGCCTGTCATAGTGATTACGGATACCACGGGCGAGGATTG |
| 47 | rcn1-PVIVIT-rev | CAATCCTCGCCCGTGGTATCCGTAATCACTATGACAGGCAAATTAGGACTGTGTC |
| 48 | pDHB1_cnA_F | ATTAACAAGGCCATTACGGCCGAAGATGGAAGTCAAGTGAGCACGATG |
| 49 | pDHB1_cnA_Rb | AACTGATTGGCCGAGGCGGCCCCCGTGCTAAGCCTTCTGGAAAGCAATTG |
| 50 | pPR3-N_CNB_F | ATTAACAAGGCCATTACGGCCATGGGAAACACAAGTAGTGCAGTATTG |
| 51 | pPR3-N_CNB_R | AACTGATTGGCCGAGGCGGCCTCAAAATTGATCTAAAGTCATACTCAT |
| 52 | pPR3-N_rcn1_F | ATTAACAAGGCCATTACGGCCATGTCATCTCCAACTTCCCCAACCGGA |
| 53 | pPR3-N_rcn1_R | AACTGATTGGCCGAGGCGGCCCTAAAACTCCATCAACTCGACCGGCGG |
